# Targeting regulatory T cells in gastrointestinal cancers: current status and future perspectives

**DOI:** 10.1007/s10147-026-02990-8

**Published:** 2026-05-11

**Authors:** Koichi Jinushi, Takuro Saito, Yuichiro Doki

**Affiliations:** 1 Department of Gastroenterological Surgery, Graduate School of Medicine, The University of Osaka, Suita, Osaka 565-0871 Japan; 2 Department of Clinical Research in Tumor Immunology, Graduate School of Medicine, The University of Osaka, 2-2 Yamada-Oka, Suita, Osaka 565-0871 Japan

**Keywords:** Regulatory T cells, Tumor immune microenvironment, Gastrointestinal cancers, Immune checkpoint inhibitors, Treg-targeted immunotherapy

## Abstract

Although immune checkpoint inhibitors (ICIs) have revolutionized the gastrointestinal cancer treatment landscape, offering durable clinical benefits in a subset of patients, their efficacy remains limited to this subset of patients, underscoring the urgent need to elucidate the mechanisms underlying immune resistance and develop novel therapeutic strategies. Increasing evidence indicates that the immunosuppressive tumor immune microenvironment (TIME) is a major obstacle to effective antitumor immunity, with regulatory T cells (Tregs) emerging as the central cellular mediators of this process. Tumor-infiltrating Tregs possess potent immunosuppressive functions and suppress cytotoxic CD8^+^ T cell-mediated responses through multiple mechanisms, including interleukin-2 consumption, cytotoxic T-lymphocyte-associated protein 4-dependent inhibitory signaling, and secretion of immunosuppressive cytokines. Recent advances in spatial transcriptomics and single-cell technologies have catalogued substantial functional heterogeneity among Tregs and revealed their preferential accumulation at tumor margins, suggesting a potential link to immune-excluded TIME phenotypes. In this review, we summarize the known Treg-mediated immunosuppressive mechanisms in gastrointestinal cancers, highlight their spatial and functional characteristics within the TIME, and discuss the status of current Treg-targeted immunotherapeutic strategies and possible future strategies to overcome ICI resistance.

## Introduction

Gastrointestinal cancers are highly prevalent worldwide, with esophageal, hepatocellular, biliary tract, and pancreatic cancers showing particularly poor prognoses [1]. Traditionally, these cancers have been treated with surgery, radiotherapy, and chemotherapy. However, these strategies offer limited benefits in patients with advanced disease [2]. Immune checkpoint inhibitors (ICIs) have recently emerged as the fourth major treatment modality and are now widely used to treat gastrointestinal cancers [3]. ICIs have been approved for several gastrointestinal malignancies, including esophageal, gastric, colorectal, hepatocellular, and biliary tract cancers [4–8], with their clinical indications continuing to expand [9–11]. ICIs enhance antitumor immunity by inhibiting immune checkpoint pathways and restoring cytotoxic T-cell functions [12, 13]. To date, they have demonstrated efficacy in limited subsets of patients. Although response rates of approximately 30% have been reported in microsatellite instability-high (MSI-H)/deficient mismatch repair (dMMR) colorectal cancers, response rates in other gastrointestinal cancers remain as low as 10–20% [4–6, 14]. Their limited population penetrance highlights the need for a deeper understanding of immune resistance and development of novel therapeutic strategies.

Tumor cells evade immune surveillance through multiple immunosuppressive mechanisms [15]. Among these, regulatory T cells (Tregs) have emerged as a key subset of immune cells capable of suppressing antitumor immunity. Tregs are a CD4^+^ T-cell population that expresses FoxP3 and exhibits potent immunosuppressive activity [16–18]. In the tumor immune microenvironment (TIME), the accumulation of activated Tregs impairs cytotoxic T-cell function, promoting immune evasion [19]. Abundant Treg infiltration is associated with poor prognosis in multiple cancer types [20]. These observations suggest that targeting Tregs represents a promising strategy for enhancing antitumor immunity. This review summarizes the immunosuppressive roles of Tregs, their involvement in tumor progression and therapeutic resistance, and the potential challenges of Treg-targeted immunotherapy in gastrointestinal cancers.

## Tumor immune microenvironment

The tumor microenvironment (TME) comprises cancer cells, immune cells, fibroblasts, vascular endothelial cells, and various cytokines and chemokines, and orchestrates interactions that shape essential tumor characteristics, including growth, invasion, metastasis, and resistance [21]. The TIME represents the immune component of the TME and plays a pivotal role in cancer development and progression. Within the TIME, antitumor immune cells, including cytotoxic T cells, natural killer (NK) cells, and dendritic cells, coexist with immunosuppressive populations, including Tregs, myeloid-derived suppressor cells (MDSCs), and tumor-associated macrophages. The balance between these opposing immune components critically influences tumor behavior and the response to treatment. The dynamic interactions between tumor cells and the immune system are conceptualized as cancer immunoediting [15], which consists of elimination, equilibrium, and escape phases. During elimination, immune surveillance suppresses tumor growth. Eventually, some tumor cells acquire resistance by downregulating antigen presentation, reducing major histocompatibility complex expression, or secreting immunosuppressive factors. These resistant clones survive and proliferate, leading to the establishment of an immunosuppressive TIME, allowing tumor escape from immune control. Based on their immunological features, TIME can be categorized into immune desert, immune-excluded, and immune-inflamed patterns [22]. Among these, the immune-inflamed phenotype, characterized by abundant CD8^+^ T-cell infiltration and high PD-L1 expression, is ICI responsive, whereas the immune-excluded and immune-desert phenotypes exhibit poor responsiveness to immunotherapy.

In gastrointestinal cancers, the TIME composition and immune evasion patterns vary with tumor type. MSI-H/dMMR colorectal cancers exhibit abundant CD8^+^ T-cell infiltration and demonstrate favorable responsiveness to ICIs [23]. In contrast, hepatocellular carcinoma, biliary tract cancer, and pancreatic cancer are characterized by abundant fibrotic stroma that restricts immune cell infiltration, resulting in the limited efficacy of ICI monotherapy [24–26]. In cancers associated with chronic inflammation or viral infection, including hepatocellular carcinoma and some gastric cancers, immunosuppressive cells, including Tregs and MDSCs, are persistently activated, contributing to immune tolerance and therapeutic resistance [27–29].

## Tregs: characteristics and clinical relevance

### Tregs in the TIME

One of the major factors limiting the breadth of cancer immunotherapy efficacy is the presence of an immunosuppressive state within the TIME, in which Tregs exert suppressive functions and serve as key mediators of tumor immune evasion. Tregs are a subset of CD4^+^ T cells characterized by high expression of the transcription factor FoxP3 [16]. Tregs play indispensable roles in maintaining immune tolerance and preventing autoimmune diseases, but constitute a major barrier to effective antitumor immunity within the tumor microenvironment [30]. Tregs suppress activation of other immune cells via secretion of immunosuppressive cytokines including transforming growth factor-β (TGF-β) and interleukin-10 (IL-10), and through cell–cell contact-dependent mechanisms [31]. Conversely, Treg inhibition induces tumor regression, highlighting their potential as promising therapeutic targets [19].

### Immunosuppressive mechanisms of Tregs (Fig. 1)

**Fig. 1:**
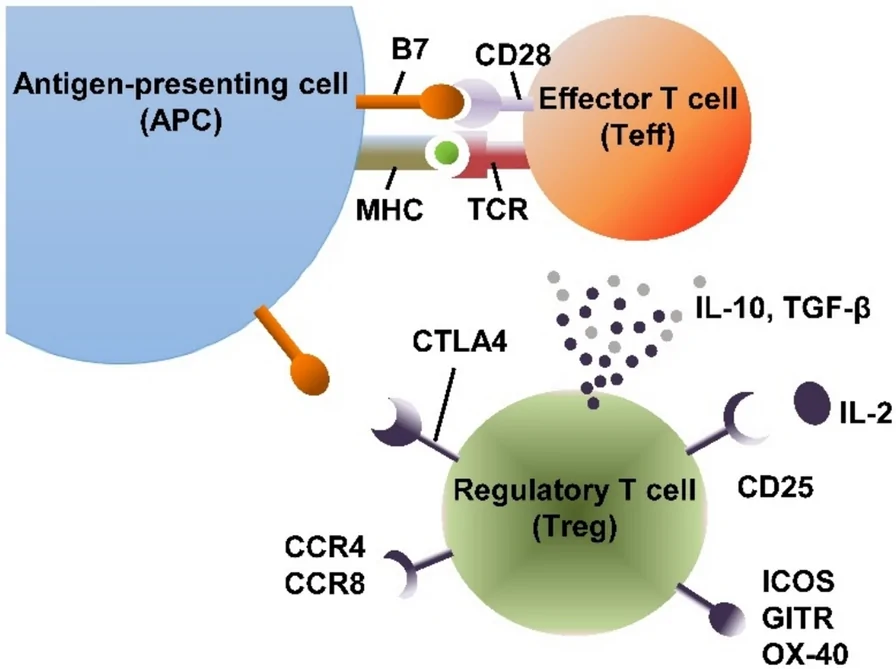
Mechanisms of effector T-cell activation and Treg-mediated immunosuppression. Antigen-presenting cells (APCs) activate effector T cells (Teff) through T-cell receptor (TCR) stimulation and the expression of costimulatory molecules. In contrast, regulatory T cells (Tregs) suppress immune responses by secreting inhibitory cytokines, including IL-10 and TGF-β, consuming IL-2 through high CD25 expression, limiting Teff proliferation, and modulating APC function via CTLA-4 to downregulate CD80/CD86 expression, thereby disrupting Teff costimulatory signaling

The immunosuppressive functions of Tregs are primarily mediated by IL-2 deprivation, cytotoxic T-lymphocyte-associated protein 4 (CTLA-4)-dependent blockade of costimulatory signaling, and secretion of inhibitory cytokines, including IL-10 and TGF-β. Although Tregs produce small amounts of IL-2, they constitutively express the high-affinity IL-2 receptor (CD25), efficiently sequestering IL-2 in their surroundings, thereby inhibiting effector T-cell activation and proliferation [32]. Tregs constitutively express CTLA-4, which suppresses CD80/CD86 expression in antigen-presenting cells through transendocytosis, leading to blockade of costimulatory signals and attenuation of T-cell activation [33, 34]. Tregs secrete immunosuppressive cytokines including TGF-β, IL-10, and IL-35, which suppress the functions of effector T cells and antigen-presenting cells, extending their suppressive effects [35, 36]. In addition to cytokine-mediated mechanisms, Tregs exert metabolic suppression by generating adenosine through ectoenzymes, including CD39 and CD73, and by transferring cyclic AMP to target cells through gap junctions, thereby inhibiting cellular functions [37, 38]. Tregs have been reported to express granzyme B and perforin, enabling them to eliminate effector T and NK cells directly through cytotoxic mechanisms [39]. Owing to their functional diversity, Tregs are critical regulators of antitumor immunity and play central roles in shaping the immunosuppressive tumor microenvironment.

### Functional heterogeneity of Tregs (Fig. 2)

**Fig. 2:**
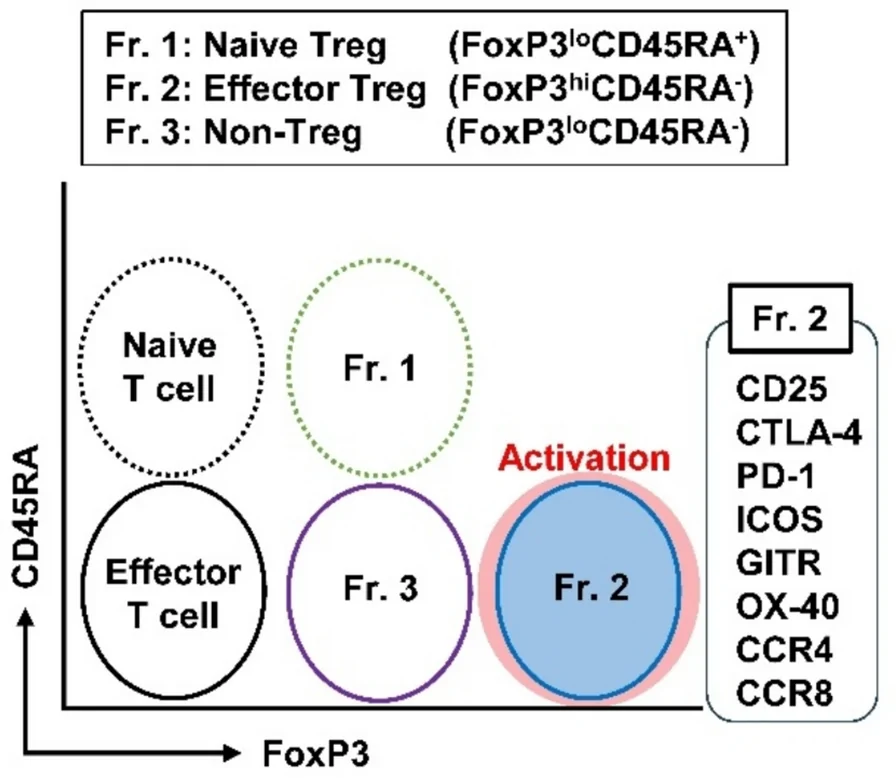
Representative regulatory T-cell (Treg) subsets. Fraction 2 (Fr.2) Tregs are characterized as effector Tregs and preferentially express immunoregulatory molecules, including CD25, CTLA-4, PD-1, ICOS, GITR, OX40, CCR4, and CCR8. These markers reflect the activated state of intratumoral Tregs and are promising targets for immunotherapy

Although tumor-infiltrating Tregs exhibit strong immunosuppressive activity, they display considerable phenotypic and functional heterogeneity. A representative classification system categorizes human Tregs into three subpopulations based on their expression levels of CD45RA and FoxP3: Fr. 1 (FoxP3^lo^CD45RA^+^ cells, naïve Tregs), Fr. 2 (FoxP3^hi^CD45RA^–^ cells, effector Tregs), and Fr. 3 (FoxP3^lo^CD45RA^–^ cells, non-Tregs) [40]., Fr. 1 and Fr. 2 cells, exhibit potent immunosuppressive activity and preferentially accumulate within tumors, thereby establishing an immunosuppressive TIME. In contrast, Fr. 3 cells lack suppressive function, instead producing proinflammatory cytokines, including IL-17 and interferon-γ. Approximately half of colorectal cancers exhibit an Fr. 3-dominant phenotype, in which inflammatory responses triggered by bacterial infiltration promote Fr. 3 cells within the TIME [41]. These findings indicate that FoxP3^+^ cells are not uniformly immunosuppressive and highlight the importance of considering the functional heterogeneity of FoxP3^+^ T-cell subsets.

### Interactions between Tregs and other immune cells

Recent advances in spatial profiling technologies, particularly spatial transcriptomics and methods integrated with single-cell analyses, have enabled the characterization of Treg spatial patterns and their interactions with neighboring immune cells. Spatial analyses following the temporal progression from colonic adenoma to colorectal carcinoma have demonstrated that Tregs preferentially accumulate at the tumor–normal tissue interface, where the Midkine–syndecan-4 signaling axis mediates their spatial organization [42]. Similarly, in renal cell carcinoma, a highly immunosuppressive subset of IL-1β⁺ Tregs localizes at the tumor margin, where they establish an immunosuppressive niche through interactions with macrophages [43]. Collectively, these findings suggest that Tregs are not uniformly distributed throughout tumors, but rather accumulate at tumor boundaries, forming a “physical and functional barrier” that restricts CD8^+^ T-cell infiltration into the tumor core. Such spatial configurations likely contribute to the development of non-inflamed TIME phenotypes.

## Therapeutic strategies targeting Tregs

Anti-PD-1 antibodies enhance antitumor immunity by reinvigorating PD-1-expressing cytotoxic CD8^+^ T cells in the TIME. PD-1 is expressed on Tregs, suggesting that anti-PD-1 therapy may counter its efficacy by activating Tregs, potentially contributing to rapid tumor progression, a phenomenon known as hyperprogressive disease (HPD) [44]. Tumors exhibiting HPD sometimes display abundant Treg infiltration, although the causal relationship remains unclear. These observations highlight the potential of selectively targeting effector Tregs to enhance antitumor immune responses. In this context, it is critical to deplete and modulate tumor-infiltrating effector Tregs while preserving CD8^+^ effector T cells, as nonspecific systemic Treg depletion carries a substantial risk of inducing severe autoimmune complications [19]. Multiple approaches to selectively target Tregs by targeting molecules preferentially expressed on Tregs have been proposed. Herein, we summarize the current status of the clinical development of representative Treg-targeted therapies, including agents directed against CD25, CTLA-4, and CCR4, as well as emerging therapeutic targets. Representative clinical trials of Treg-targeted therapies for gastrointestinal cancers are summarized in Table 1.

**Table 1:** Clinical trials of Treg-targeted therapies in gastrointestinal cancers

Target	Agent/code	Trial ID	Cancer type	Phase	Design/combination	Primary endopoint	Status
CD25	RG6292	NCT04158583	Advanced/metastatic solid tumors (incl. esophageal cancer)	I	Mono	Safety, tolerability, MTD, RP2D	Completed
CD25	RG6292	NCT04642365	Advanced/metastatic solid tumors	Ib	RG6292 + Atezo	TEAE, DLT, ORR	Terminated
CD25	ADCT-301	NCT03621982	Advanced solid tumors	Ib	Mono/± Pembro	TEAE, DLT	Terminated
CD25	RM-1995	jRCT2031220721	Advanced/recurrent solid tumors with liver metastases	I	Mono/± Pembro	Safety, tolerability, MTD, RP2D	Recruiting
CTLA4	Ipilimumab	NCT02872116	Advanced/metastatic gastric/gastroesophageal junction/esophageal cancer	III	Nivo + Chemo/Nivo + Ipi/Chemo	OS (CPS ≥ 5)	Completed
**CTLA4**	**Ipilimumab**	**NCT03143153**	**Advanced/metastatic esophageal squamous cell carcinoma**	**III**	**Nivo + Chemo/Nivo + Ipi/Chemo**	**OS, PFS**	**Completed**
**CTLA4**	**Tremelimumab**	**NCT03298451**	**Unresectable hepatocellular carcinoma**	**III**	**Treme + Durva/Durva/Sora**	**OS**	**Active, not recruiting**
**CTLA4**	**Ipilimumab**	**NCT04008030**	**Advanced/metastatic MSI-H/dMMR colorectal cancer**	**III**	**Nivo + Ipi/Nivo/Chemo**	**PFS**	**Active, not recruiting**
**CTLA4**	**Ipilimumab**	**NCT04039607**	**Advanced hepatocellular carcinoma**	**III**	**Nivo + Ipi/Sora/Len**	**OS**	**Active, not recruiting**
CTLA4	Tremelimumab	NCT05301842	Locoregional hepatocellular carcinoma	III	TACE + Durva/TACE + Durva + Treme ± Len	OS, PFS, ORR	Active, not recruiting
CCR4	Mogamulizumab	NCT01929486	Advanced solid tumors (incl. esophageal, gastric cancer)	Ib	Mono	Safety, eTreg depletion in PBMCs	Completed
CCR4	Mogamulizumab	NCT02281409	Advanced and/or metastatic solid tumors (incl. gastric cancer)	I/II	Mono	MTD	Completed
CCR4	Mogamulizumab	NCT02301130	Advanced solid tumors (incl. pancreatic and colorectal cancer)	I	Moga + Durva/Moga + Treme	TEAE, DLT	Completed
CCR4	Mogamulizumab	NCT02476123	Advanced/metastatic solid tumors (incl. esophageal, colorectal, pancreatic, and hepatocellular carcinoma)	I	Moga + Nivo	TEAE, DLT	Completed
CCR4	Mogamulizumab	NCT02705105	Advanced/metastatic solid tumors (incl. esophageal, colorectal, pancreatic and hepatocellular carcinoma)	I/II	Moga + Nivo	Safety, tolerability, MTD, RP2D	Completed
CCR4	Mogamulizumab	NCT02946671	Operable solid tumors (incl. esophageal cancer)	I	Moga + Nivo (neoadjuvant)	Safety, intratumoral Treg depletion	Completed
CCR8	denikitug	NCT05007782	Advanced solid tumors	I	Mono/± Zim	DLT, TEAE	Recruiting
CCR8	S-531011	NCT05101070	Locally advanced or metastatic solid tumors	Ib/II	Mono/± Pembro	TEAE, ORR, PFS、OS	Recruiting
CCR8	LM-108	NCT05518045	Advanced solid tumors	I/II	Mono/± Tori	TEAE, DLT	Active, not recruiting
CCR8	BAY3375968	NCT05537740	Advanced solid tumors	I	Mono/± Pembro	TEAE, MTD, DLT	Active, not recruiting
CCR8	CHS-114	NCT05635643	Advanced solid tumors	I	Mono/± Tori	DLT, Safety, tolerability	Recruiting
CCR8	BGB-A3055	NCT05935098	Advanced or metastatic solid tumors	I	Mono/± Tisle (± Chemo)	TEAE, MTD, DLT, ORR	Active, not recruiting
CCR8	AMG 355	NCT06131398	Advanced solid tumors	I	Mono/± Pembro	DLT, TEAE	Recruiting
CCR8	LM-108	NCT06873854	Advanced solid tumours (MSI-H/dMMR advanced solid tumours)	II	LM-108 + Tori	ORR	Not yet recruiting
CCR8	CHS-114	NCT06657144	Advanced or metastatic solid tumors	I	CHS-114 + Tori (± Chemo)	TEAE	Recruiting
CCR8	DT-7012	NCT06819735	Advanced solid tumors	I/II	Mono/± ICI	DLT, TEAE	Recruiting
CCR8	TAK-188	NCT07205718	Advanced or spreading solid tumors	I/II	Mono	TEAE, DLT, ORR	Not yet recruiting
ICOS	Alomfilimab	NCT03829501	Advanced solid tumors (incl. esophageal, colorectal, pancreatic and hepatocellular carcinoma)	I/II	Mono/± Atezo	Safety, tolerability, MTD, RP2D, ORR	Completed

CTLA4, cytotoxic T-lymphocyte antigen 4; CCR4: CC motif chemokine receptor 4; ICOS, inducible T-cell costimulator; CCR8, CC motif chemokine receptor 8; MSI-H, microsatellite instability-high; dMMR, deficient mismatch repair; Mono, monotherapy; Atezo, atezolizumab; Pembro, pembrolizumab; Nivo, nivolumab; Chemo, chemotherapy; Ipi, ipilimumab; Treme, tremelimumab; Durva, durvalumab; Sora, sorafenib; Len, lenvatinib; TACE, transcatheter arterial chemoembolization; Moga, mogamulizumab; Zim, zimberelimab; Tori, toripalimab; Tisle, tislelizumab; ICI, immune checkpoint inhibitor; MTD, maximum tolerated dose; RP2D, recommended phase 2 dose; TEAE = treatment-emergent adverse event; DLT, dose-limiting toxicity; ORR, overall response rate; OS, overall survival; CPS, combined positive score; PFS, progression-free survival; Treg, regulatory T cell; PBMC, peripheral blood mononuclear cells

Regimens currently approved and used in routine clinical practice are shown in bold

### CD25-targeted therapy

CD25 was the first surface marker used to identify and isolate Tregs, preceding the discovery of the master transcription factor, FoxP3, and was the earliest target for Treg depletion strategies [17]. Early clinical trials targeting CD25 investigated daclizumab (a humanized IgG1 anti-human CD25 monoclonal antibody) and denileukin diftitox (a recombinant fusion protein composed of IL-2 and a diphtheria toxin fragment), often in combination with cancer vaccines [45–48]. CD25 is not Treg specific and is transiently expressed on activated CD8^+^ and CD4^+^ T cells, which risks concomitant effector T-cell suppression. None of these trials demonstrated a survival benefit, and no CD25-targeted agent has been approved for anti-Treg therapy. Near-infrared photoimmunotherapy (NIR-PIT) has recently emerged as a promising approach for spatially selective depletion of Tregs. NIR-PIT uses an antibody–photoabsorber conjugate that links an anti-CD25 antibody to the photosensitive dye IR700 (IRDye700DX). Upon local irradiation with near-infrared light (~ 690 nm), this conjugate induces irreversible membrane damage, leading to selective cell death [49]. This strategy selectively eliminates CD25-high Tregs within tumors, while preserving peripheral Tregs and promoting CD8^+^ T and NK cell activation, enabling local immune remodeling. A Phase I clinical trial is ongoing in patients with advanced or recurrent solid tumors and liver metastases, including gastrointestinal cancer (jRCT2031220721).

### CTLA-4-targeted therapy

CTLA-4 is an immunosuppressive coinhibitory molecule highly expressed in Tregs and serves as an essential mediator of their suppressive functions. Tregs engage CD80/CD86 on antigen-presenting cells via CTLA-4, thereby competitively inhibiting CD28-mediated costimulatory signaling and suppressing CD8^+^ and CD4^+^ effector T-cell activation [50]. Based on this mechanism, CTLA-4 is an attractive therapeutic target for Treg inhibition. In Japan, ipilimumab (a fully human IgG1 anti-CTLA-4 monoclonal antibody) has been approved as a monotherapy for malignant melanoma and in combination with other agents for several malignancies, including renal cell carcinoma, MSI-H colorectal cancer (NCT02060188), non-small cell lung cancer, malignant pleural mesothelioma, esophageal cancer (NCT03143153), and hepatocellular carcinoma (NCT01658878). Although ipilimumab was initially developed to trigger inhibitory signals from T cells, recent preclinical studies have suggested that its ability to mediate antibody-dependent cellular cytotoxicity (ADCC)-dependent depletion of tumor-infiltrating Tregs may contribute to its antitumor activity [51]. As CTLA-4 is expressed on activated effector T cells, anti-CTLA-4 therapy may affect other immune cells. Non-tumor-specific Treg depletion and unintended modulation of effector T cells may underlie the relatively high incidence of immune-related adverse events associated with this class of agents [52]. Next-generation anti-CTLA-4 antibodies incorporating Fc engineering strategies are currently being developed to maximize tumor-specific Treg depletion and minimize systemic toxicity [53, 54].

### CC chemokine receptor 4 (CCR4)-targeted therapy

CCR4 is a highly expressed chemokine receptor on effector Tregs that exhibits potent immunosuppressive activity and is enriched in the TIME [55]. Thus, targeting CCR4 is a promising approach for tumor-selective Treg depletion. Mogamulizumab, an anti-CCR4 monoclonal antibody, was originally developed to treat adult T-cell leukemia/lymphoma. Owing to its potent ADCC activity, its potential to deplete Tregs from solid tumors has been extensively explored [56]. Peripheral blood Treg depletion has been observed in clinical studies of mogamulizumab. These effects are accompanied by concomitant reductions in central memory T cells, resulting in limited clinical efficacy [57–59]. CCR4 is expressed on T helper type 2 cells, and its blockade can disrupt immune homeostasis in the skin, leading to increased production of inflammatory cytokines and activation of epidermotropic CD8^+^ T cells, ultimately causing clinically significant cutaneous toxicity [60]. Although clinical trials combining mogamulizumab and nivolumab in solid tumors, including esophageal, colorectal, pancreatic, and hepatocellular carcinomas, have demonstrated a substantial reduction in CCR4^+^ Tregs within tumors and enhanced T-cell infiltration into the TIME [61, 62], the overall therapeutic efficacy remains limited.

### CC chemokine receptor 8 (CCR8)-targeted therapy

CCR8 is selectively expressed on effector Tregs within the TIME and exhibits minimal expression in other T-cell populations [63–65]. Preclinical studies have shown that anti-CCR8 antibodies can selectively deplete intratumoral Tregs, promote CD8⁺ T-cell infiltration into tumors, and enhance their cytokine-producing ability [66, 67]. Because CCR8 expression in peripheral tissues is extremely low, this strategy is expected to preserve normal tissue-resident Tregs while selectively relieving immunosuppression within tumors, suggesting a potentially favorable safety profile. Several clinical trials evaluating anti-CCR8 antibodies are currently underway.

### Inducible T-cell costimulator (ICOS)-targeted therapy

ICOS is a costimulatory molecule that is upregulated by T-cell receptor stimulation. In gastrointestinal malignancies, including gastric cancer and hepatocellular carcinoma, high ICOS expression has been observed in tumor-infiltrating Tregs [29, 68]. ICOS expression can be found on activated CD4^+^ effector T cells and, to a lesser extent, on subsets of CD8^+^ T cells, indicating that it is not strictly a Treg-specific target. Recently developed ICOS-targeting antibodies are IgG1 isotype molecules with ADCC capability that exhibit a dual mechanism of action, depending on antigen density [69]. In cells with high ICOS expression, particularly Tregs, these antibodies induce Fc receptor-mediated depletion, whereas in cells with relatively low ICOS levels, including CD8^+^ T cells, they provide costimulatory signals that enhance cytokine production. Preclinical studies have demonstrated that these antibodies increase the number of intratumoral Tregs and enhance CD8^+^ T-cell effector functions, resulting in synergistic antitumor activity when combined with ICIs [69]. Conversely, the ability to activate CD8^+^ T cells through costimulatory signaling may lead to off-target effects on non-Treg T-cell populations at higher doses, raising concerns regarding therapeutic selectivity and underscoring the need for careful evaluation in clinical development. Phase I/II clinical trials targeting advanced cancers, including gastrointestinal malignancies, are ongoing (NCT03829501).

### Others

Several other molecules have been explored as potential targets for Treg cell-directed therapies. Costimulatory molecules, including glucocorticoid-induced TNF receptor-related protein and OX40, are expressed on Tregs and effector T cells. Targeting these molecules under certain conditions has been suggested to modulate or attenuate Treg suppressive functions [70]. Careful selection of therapeutic targets that optimize Treg specificity, safety, and clinical applicability is essential for the development of effective Treg-targeted immunotherapies.

## Conclusions

We summarize the immunosuppressive roles of Tregs and the current landscape of Treg-targeted immunotherapies for gastrointestinal cancers. Tregs constitute a major barrier limiting ICI efficacy, and their functional heterogeneity and spatial localization within the TIME pose challenges and opportunities for therapeutic interventions. The development of selective and safe strategies that consider these characteristics is critical for enhancing antitumor immunity. Therefore, Treg-targeted approaches are expected to expand and improve the treatment options for patients with gastrointestinal malignancies.
